# *miR-31*-*NUMB* Cascade Modulates Monocarboxylate Transporters to Increase Oncogenicity and Lactate Production of Oral Carcinoma Cells

**DOI:** 10.3390/ijms222111731

**Published:** 2021-10-29

**Authors:** Chung-Hsien Chou, Chun-Yu Fan Chiang, Cheng-Chieh Yang, Ying-Chieh Liu, Sih-Rou Chang, Kuo-Wei Chang, Shu-Chun Lin

**Affiliations:** 1 Institute of Oral Biology, College of Dentistry, National Yang Ming Chiao Tung University, Taipei 112, Taiwan; michaelchou0806@gmail.com (C.-H.C.); taroislove@hotmail.com (C.-Y.F.C.); ccyang@ym.edu.tw (C.-C.Y.); yingchieh12@gmail.com (Y.-C.L.); s4103052122@gmail.com (S.-R.C.); 2 Department of Dentistry, College of Dentistry, National Yang Ming Chiao Tung University, Taipei 112, Taiwan; 3 Department of Stomatology, Taipei Veterans General Hospital, Taipei 112, Taiwan

**Keywords:** CRISPR, lactate, *MCT1*, *MCT4*, *miR-31*, *NUMB*

## Abstract

Oral squamous cell carcinoma (OSCC) is among the leading causes of cancer-associated death worldwide. *miR-31* is an oncogenic miRNA in OSCC. NUMB is an adaptor protein capable of suppressing malignant transformation. Disruption of the *miR-31*-*NUMB* regulatory axis has been demonstrated in malignancies. Mitochondrial dysfunction and adaptation to glycolytic respiration are frequent events in malignancies. Monocarboxylate transporters (MCTs) function to facilitate lactate flux in highly glycolytic cells. Upregulation of *MCT1* and *MCT4* has been shown to be a prognostic factor of OSCC. Here, we reported that *miR-31-NUMB* can modulate glycolysis in OSCC. Using the CRISPR/Cas9 gene editing strategy, we identified increases in oncogenic phenotypes, *MCT1* and *MCT4* expression, lactate production, and glycolytic respiration in *NUMB*-deleted OSCC subclones. Transfection of the *Numb1* or *Numb4* isoform reversed the oncogenic induction elicited by *NUMB* deletion. This study also showed, for the first time, that NUMB4 binds MCT1 and MCT4 and that this binding increases their ubiquitination, which may decrease their abundance in cell lysates. The disruptions in oncogenicity and metabolism associated with *miR-31* deletion and *NUMB* deletion were partially rescued by *MCT1*/*MCT4* expression or knockdown. This study demonstrated that NUMB is a novel binding partner of MCT1 and MCT4 and that the *miR-31*-*NUMB*-*MCT1/MCT4* regulatory cascade is present in oral carcinoma.

## 1. Introduction

Oral squamous cell carcinoma (OSCC) is one of the most prevalent head and neck SCCs (HNSCCs) worldwide. miRNAs are noncoding RNAs that play various important roles during pathogenesis by specifically targeting transcripts and regulating their expression levels [1,2]. *miR-31* has been shown to be an oncogenic miRNA upregulated in OSCC that modulates hypoxia, DNA repair, stemness, and various metabolic pathways [2,3,4,5,6,7,8]. *miR-31* targets *SIRT3* [7], *ACOX1* [8], *FIH* [9,10], and *SDHA* [11] to disrupt metabolic homeostasis in malignancies. However, to develop therapeutic strategies, the functions of miRNAs in regulating the metabolism of OSCC must be elucidated.

NUMB is an adaptor protein that plays multifaceted roles in modulating cell functions, including neurogenesis, symmetric cell division in the stemness process, epithelial-mesenchymal transition, and oncogenesis [12,13,14,15]. NUMB participates in the process of ubiquitin degradation by binding with ubiquitin ligase Itch to breakdown Notch or Gli [16,17,18]. In addition, NUMB also interacts with p53, MDM2, and PTEN to modulate their activity in the neoplastic process [14,16,19,20,21,22]. *miR-31* targets *NUMB* to enhance oncogenicity in OSCC and colorectal carcinoma [23,24]. In addition, *miR-96*, *miR-146a*, *miR-182*, and *miR-545* also target *NUMB* to augment oncogenicity [25,26]. The suppressor roles of *NUMB* against OSCC have been identified in previous studies [14,23], but its downstream effectors remain to be elucidated. The distinct functions mediated by six *NUMB* isoforms resulting from alternative splicing have been actively investigated [12,27]. We discovered that the *NUMB* isoforms *Numb1* to *Numb4* were capable of suppressing OSCC pathogenesis [23]. Interestingly, a recent study revealed that Numb1, but not Numb4, binds the mGluR5α glutamate receptor to inhibit its internalization in 293T cells [28]. Whether NUMB variants differentially modulate downstream effectors requires further investigation.

Metabolic symbiosis between oxidative and glycolytic cells is crucial for sustained tumor growth and metastasis. Crosstalk between SLC2A glucose transporter (GLUT) family members and SCL16A monocarboxylate transporter (MCT) family members is responsible for substrate transport in metabolic processing [29,30]. Like in many other types of malignancies, H^+^-linked lactate transporters MCT1 (SLC16A1) and MCT4 (SLC16A3), which shuttle lactate along the H^+^ gradients, have been relatively well investigated compared with other MCTs in HNSCC [29,31,32,33]. Upregulation of *MCT1* and/or *MCT4* expression has also been shown to be a prognostic marker of HNSCC [34,35]. Complex mechanisms including transcriptional modulation, miRNA targeting, post-translational modifications, and others may be involved in regulating the expression and functions of *MCT1*/*MCT4* [30]. The present study provided novel clues demonstrating that NUMB binds MCT1/MCT4 to induce MCT1/MCT4 degradation, decrease oncogenicity, and increase glycolytic metabolism in OSCC cells. The *miR-31*-*NUMB*-*MCT1*/*MCT4* regulatory axis appears to be an important therapeutic target in OSCC.

## 2. Results

### 2.1. Establishment of miR-31 Deletion Subclones

**Figure 1:**
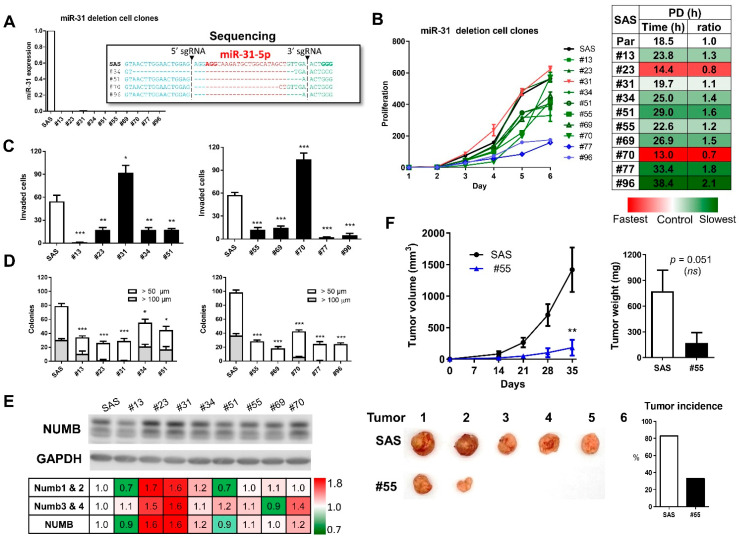
*miR-31* deletion suppresses OSCC oncogenicity. (**A**) qRT-PCR analysis of *miR-31* expression in representative subclones revealed the complete absence of *miR-31* expression. Representative sequencing results, shown in the indent, demonstrated the truncation of *miR-31*-5p segments in the subclones. *miR-31* expression levels and the truncation of *miR-31*-5p in other subclones are shown in Figure S1B,C. (**B**,**C**) Growth and invasion assays. The right panel of (**B**) is a heatmap illustrating the population doubling time of subclones. Growth or invasion was reduced in 8 of the 10 subclones. PD, population doubling time (h). (**D**) Anchorage-independent colony formation. Anchorage-independent growth was decreased in all subclones. (**E**) Upper panel, Western blot analysis of NUMB expression in subclones relative to parental cells. Lower panel, heatmap illustrating the expression levels. Upper bands, the signals of Numb1 and Numb 2 isoforms. Lower bands, the signals of Numb3 and Numb4 isoforms. *NUMB* signals were increased in the analyzed subclones except for #13 and #51. (**F**) Tumorigenicity of subclone #55. Upper left panel, the growth curve measured weekly. Upper right panel, the tumor weight at week 5. Lower left panel, the images of harvested tumors. Lower right panel, the incidence of tumor induction. *ns*, not significant; *, *p* < 0.05; **, *p* < 0.01; ***, *p* < 0.001.

The strategy used for deleting the *miR-31* gene by means of the CRISPR/Cas9 system is shown in Figure S1A. Cleavage of Cas9 at 5′ sgRNA and 3′ sgRNA cut sites should result in *miR-31* gene truncation. SAS cells were selected with puromycin to obtain a total of 19 subclones lacking *miR-31* expression (Figure S1B). Sequencing of 10 subclones confirmed the deletion of nucleotides of various lengths between cut sites (Figure S1C). Deletion subclones were subjected to phenotypic analysis (Figure 1A). The results showed growth inhibition in the majority of subclones except for #23, #31, and #70 (Figure 1B) and reduced invasion except for subclones #31 and #70 (Figure 1C). The colony formation capability was impaired in all tested subclones (Figure 1D). The expression of NUMB isoforms in deletion subclones were upregulated to varying extents. The increases in #23, #31, #34, #55, and #70 were modest (Figure 1E). Subclones #13 and #51 had slight decreases in the Numb1 and Numb 2 isoforms. The subcutaneous tumorigenicity of subclone #55 in nude mice was significantly attenuated (Figure 1F). Thus, we concluded that the *miR-31* gene locus was required to elicit oncogenesis in OSCC cells.

### 2.2. Establishment of NUMB Deletion and Activation Systems

**Figure 2:**
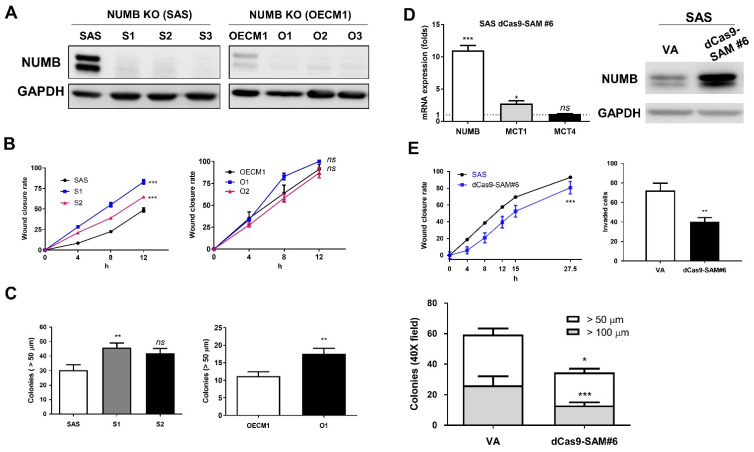
*NUMB* deletion increases the OSCC oncogenicity. (**A**) Western blot analysis to detect the near absence of NUMB signals in the S1–S3 subclones of SAS cells and O1–O3 subclones of OECM1 cells compared to parental cells. (**B**) Wound healing assay. There was a conspicuous increase in wound closure in the S1 and S2 subclones but no changes in the O1 and O2 subclones. (**C**) Anchorage-independent colony formation. It shows the increased colony formation in S1, S2, and O1 subclones relative to controls. (**D**) The induction of endogenous NUMB expression using the *NUMB* dCas9-SAM system in SAS cells. The delivery of sgRNA#6 to transactivate the *NUMB* promoter increased *NUMB* mRNA (left panel) and protein (right panel) expression. (**E**) Elevated *NUMB* expression from the endogenous locus decreased the wound closure rate (upper left panel), invasion (upper right panel), and colony formation (lower panel) of SAS cells. VA, vector alone. *ns*, not significant; *, *p* < 0.05; **, *p* < 0.01; ***, *p* < 0.001.

The strategy used for deleting *NUMB* by means of the CRISPR/Cas9 system and a single sgRNA in OSCC cells is shown in Figure S2A. Sequencing of the established subclones of SAS and OECM1 confirmed the introduction of various Indel nucleotides which resulted in the frameshift and the occurrence of premature stop codons (Figure S2B). Western blot analysis revealed the nearly complete disappearance of NUMB protein expression in S1–S3 subclones established from SAS cells and O1–O3 subclones from OECM1 cells (Figure 2A). In general, these subclones exhibited increased competence in wound healing (Figure 2B) and colony formation (Figure 2C).

A synergistic activation mediator (SAM) activation strategy was adopted to increase endogenous *NUMB* expression by promoter transactivation in SAS cells (Figure S3). Among the eight potential sites used to test SAM activity, robust upregulation of *NUMB* mRNA and protein expression was achieved by dCas9-SAM#6 in SAS cells but not in unstimulated parental cells (Figure 2D). The upregulation of endogenous *NUMB* expression was significantly associated with decreased wound healing, invasion, and colony formation of SAS cells (Figure 2E).

### 2.3. Numb1 or Numb4 Expression Represses the Colony Formation of NUMB Deletion Subclones

**Figure 3:**
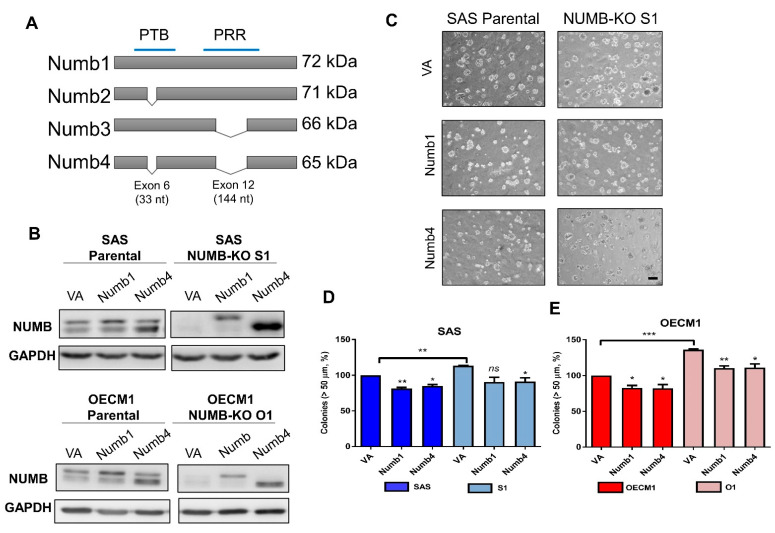
Exogenous expression of Numb1 and Numb4 isoforms increases colony formation in *NUMB*-deleted OSCC cells. (**A**) Schematic diagram illustrating the differences between *NUMB* isoforms. (**B**) Western blot analysis. This indicates differential expression of exogenous Numb1 or Numb4 in SAS, S1, OECM1, and O1 cells. (**C**) Representative photomicrographs of anchorage-independent colonies of SAS cells following *NUMB* knockout or the exogenous expression of Numb1 or Numb4. Bar, 200 µM. (**D**) Quantitation of colonies in SAS cells (left) and (**E**) OECM1 cells (right). Percentage grading was used to integrate three or four individual assays. VA, vector alone. *ns*, not significant; *, *p* < 0.05; **, *p* < 0.01; ***, *p* < 0.001.

The structures of *Numb1*–*Numb4* isoforms frequently present in cells are illustrated in Figure 3A. Compared to *Numb1,* which is the complete *NUMB* isoform, the *Numb4* isoform lacks 59 residues in functional domains PTB and PRR and its molecular weight is approximately 7 kDa smaller. Parental SAS and OECM1 cells and S1 and O1 subclones were transfected with *Numb1* and *Numb4* isoform constructs which we previously established [23]. Western blot analysis revealed the differential expression of these proteins in cells (Figure 3B). Colony formation assays indicated that either *Numb1* or *Numb4* expression repressed colony formation in SAS or OECM1 cells (Figure 3C–E). The increased colony formation phenotype of *NUMB*-deleted subclones S1 and O1 was also reversed by the expression of either *Numb1* or *Numb4*. Altogether, we concluded that *Numb1* and *Numb4* isoforms resulted in comparable reductions in colony formation in OSCC cells.

### 2.4. Reduced NUMB Expression Upregulates MCT1/MCT4 Expression and Glycolytic Respiration

**Figure 4:**
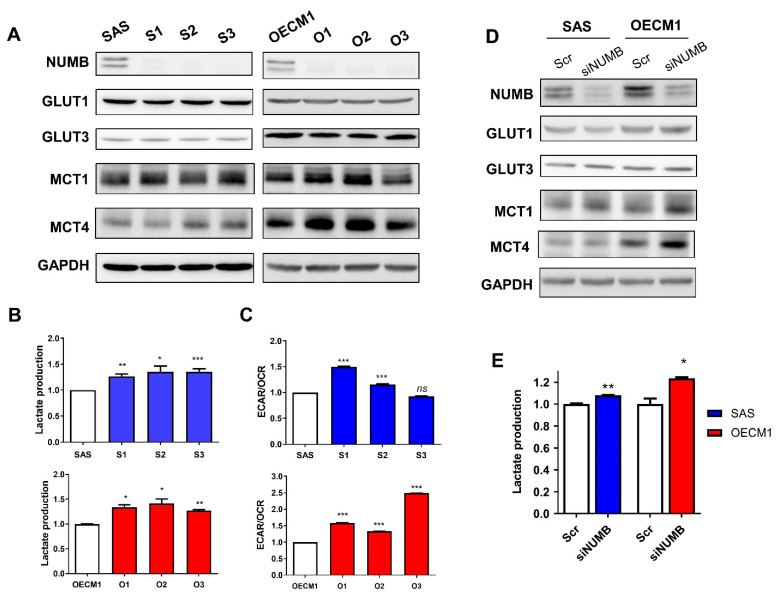
*NUMB* deletion or knockdown increases the expression of MCT1/MCT4 and glycolysis in OSCC cells. (**A**,**D**) Western blot analysis of knockout subclones and knockdown cells. There was a general tendency towards increased MCT1 and MCT4 expression in *NUMB*-deleted or NUMB-knockdown SAS and OECM1 cells. The expression of GLUT1 and GLUT3 was not changed. (**B**,**E**) Lactate production assay of knockout subclones and knockdown cells. The *NUMB-*deleted subclones and knockdown cells exhibited increased lactate production. (**C**) The anaerobic respiration shown by the ECAR/OCR ratio, which was measured by Seahorse flux analysis, increased in most *NUMB* knockout subclones. Scr, scramble oligonucleotide. *ns*, not significant; *, *p* < 0.05; **, *p* < 0.01; ***, *p* < 0.001.

Western blot analysis indicated the upregulation of MCT1 in S1, S3, O1, and O2 subclones relative to parental cells and the upregulation of MCT4 in S2, S3, O1, O2, and O3 subclones relative to parental cells (Figure 4A). However, GLUT1 and GLUT3 expression in OSCC cells was not changed following the deletion of *NUMB*. Lactate production (Figure 4B) and anaerobic respiration (Figure 4C) increased in *NUMB*-deleted subclones. To confirm the effects of *NUMB*, knockdown of *NUMB* was carried out in OSCC cells. We found that MCT1/MCT4 expression (Figure 4D) and lactate production (Figure 4E) increased when *NUMB* was knocked down in SAS and OECM1 cells.

### 2.5. MCT1 and MCT4 Increase the Oncogenicity of OSCC Cells

**Figure 5:**
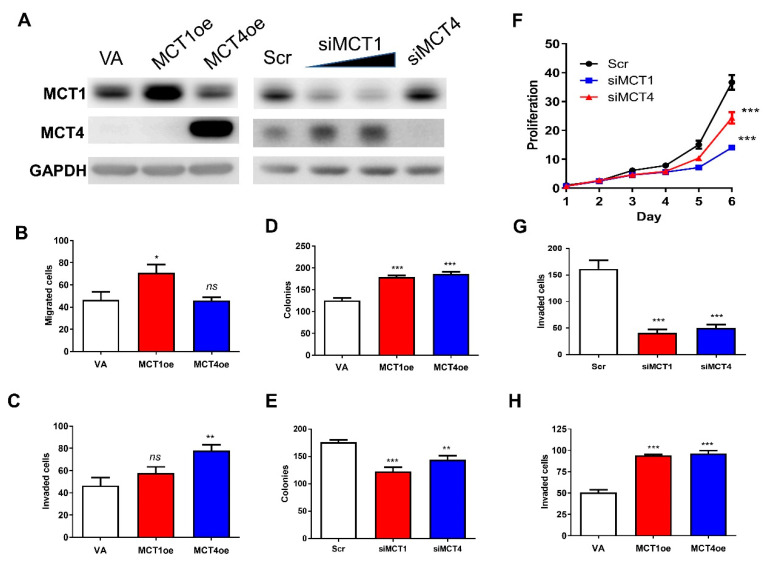
Association between MCT1/MCT4 expression and OSCC oncogenicity. (**A**–**E**) SAS cells. (**F**–**H**) OECM1 cells. (**A**) Western blot analysis to confirm the exogenous expression or knockdown of MCT1 or MCT4 in SAS cells. The signal intensity of MCT4 image in left panel was attenuated to acquire better illustration. Regulation between MCT1 and MCT4 seemed to exist. (**B**) Migration assay. (**C**,**G**,**H**) Invasion assay. (**D**,**E**) Anchorage-independent colony formation. (**F**) Proliferation. (**B**–**D**) Exogenous MCT1 or MCT4 expression generally increased the migration, invasion and colony formation of SAS cells. (**E**) Knockdown of MCT1 or MCT4 significantly decreased colony formation in SAS cells. (**F**) Knockdown of MCT1 or MCT4 decreased the proliferation of OECM1 cells. (**G**,**H**) Knockdown of MCT1 or MCT4 decreased invasion, while exogenous expression increased the invasion of OECM1 cells. Scr, scramble oligonucleotide; VA, vector alone. *ns*, not significant; *, *p* < 0.05; **, *p* < 0.01; ***, *p* < 0.001.

Exogenous expression and knockdown of MCT1/MCT4 were carried out in SAS and OECM1 cells. Western blot analysis confirmed exogenous expression and knockdown in SAS cells (Figure 5A). In SAS cells, exogenous MCT1 expression increased migration (Figure 5B), while exogenous MCT4 expression increased invasion (Figure 5C). Both MCT1 and MCT4 increased colony formation when expressed exogenously (Figure 5D). The knockdown of MCT1 or MCT4 expression drastically decreased colony formation (Figure 5E). In OECM1 cells, knockdown of MCT1 or MCT4 expression markedly decreased growth and invasion (Figure 5F,G). Exogenous MCT1 or MCT4 expression increased the invasion of OECM1 cells (Figure 5H). In summary, the results suggest that MCT1 and MCT4 enhance oncogenicity in OSCC cells. In the GSE37991 Gene Expression Omnibus (GEO) database, upregulation of both *MCT1* and *MCT4* was noted in OSCC tumors (Figure S4A). Although the correlation between the expression of *MCT1* and *MCT4* is lacking, the vast majority (75%) of tumors upregulated *MCT1* and *MCT4* simultaneously. In The Cancer Genome Atlas (TCGA) HNSCC dataset, a significant correlation between the expression of both *MCT1* and *MCT4* was found (Figure S4B). In addition, higher *MCT1* or *MCT4* expression in tumors was associated with the worse survival of patients. Tumors exhibiting high expression of both *MCT1* and *MCT4* displayed even worse patient survival. Thus, oncogenic roles of *MCT1* and *MCT4* are supported by the findings in patient-derived tissues.

### 2.6. NUMB4 Binds MCT1/MCT4 and Induces Polyubiquitination

**Figure 6:**
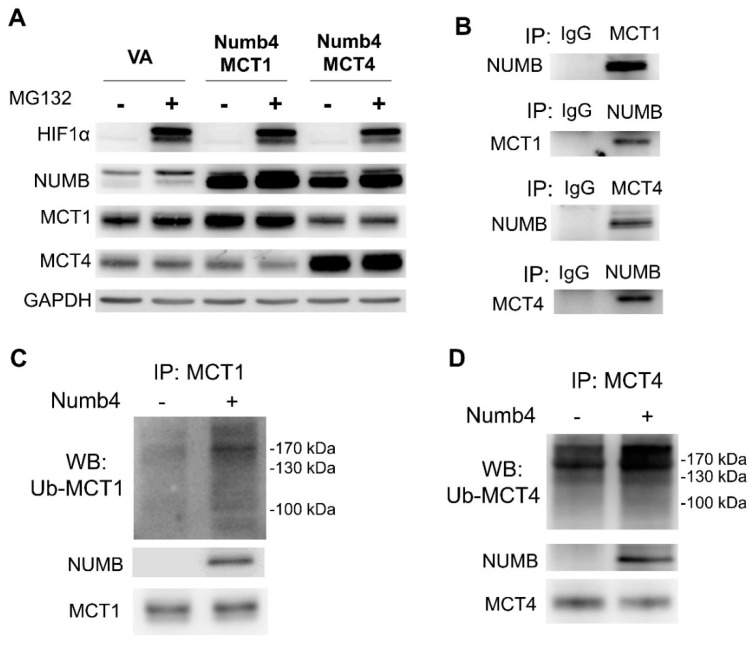
Numb4 binds MCT1 and MCT4 and facilitates their polyubiquitination. (**A**) Western blot analysis of 293T cells transfected with Numb4, MCT1, and MCT4 constructs and treated with MG132. HIF1α expression serves as a control to validate the efficacy of MG132. Treatment with 20 µM MG132 for 8 h increased the abundance of endogenous and exogenous NUMB, but it did not consistently affect exogenous or endogenous MCT1 or MCT4. Exogenous MCT4 expression decreased the abundance of endogenous MCT1. (**B**) Immunoprecipitation. Immunoprecipitates generated by anti-NUMB antibody contained MCT1 and MCT4 in 293T cells. Immunoprecipitates generated by anti-MCT1 or anti-MCT4 antibody also contained NUMB. (**C**) Enhanced polyubiquitination of MCT1 in the immunoprecipitates generated by anti-MCT1 following Numb4 expression in SAS cells. (**D**) Enhanced polyubiquitination of MCT4 in the immunoprecipitates generated by anti-MCT4 following Numb4 expression in 293T cells. VA, vector alone.

Treatment of 293T cells with MG132 increased the abundance of HIF1α and NUMB, probably due to the inhibition of proteasomal activity (Figure 6A). Numb4, MCT1, or MCT4 were exogenously expressed beyond the endogenous levels when appropriate plasmids were transfected either by themselves or in combination. The net changes in MCT1 and MCT4 following treatment are difficult to evaluate since HIF1α, NUMB, and MG132 can regulate MCT1/MCT4 expression [36]. In 293T cells co-expressing Numb4 and MCT1, the analysis of immunoprecipitates isolated by anti-NUMB antibody or anti-MCT1 antibody detected signals of MCT1 and NUMB, respectively (Figure 6B, upper panels). In cells co-expressing Numb4 and MCT4, the analysis of immunoprecipitates isolated by anti-NUMB antibody or anti-MCT4 antibody detected signals of MCT4 and NUMB, respectively (Figure 6B, lower panels). The results indicate that Numb4 can bind MCT1 and MCT4. In the immunoprecipitates isolated by anti-MCT1 antibody in SAS cells expressing ubiquitin and Numb4, polyubiquitination of MCT1, and co-precipitated NUMB increased (Figure 6C). In the immunoprecipitates isolated by anti-MCT4 antibody in 293T cells expressing ubiquitin and Numb4, polyubiquitination of MCT4, and NUMB increased and MCT4 decreased (Figure 6D). These observations indicate that Numb4 is involved in the ubiquitination of MCT1 and MCT4.

### 2.7. miR-31- and NUMB-Deletion Phenotypes Are Reversed by Modulating MCT1/MCT4 Expression

**Figure 7:**
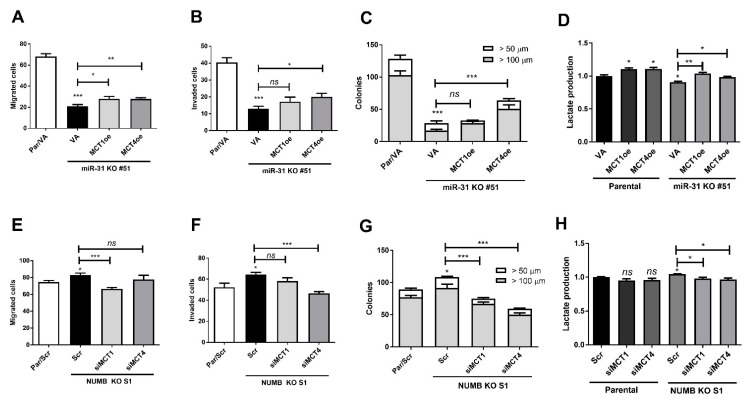
Reversal of *miR-31*-*NUMB*-associated tumor phenotypes and lactate production by *MCT1* and *MCT4*. (**A**–**D**) SAS cell and SAS *miR-31* KO#51 subclone. (**E**–**H**) SAS cells and SAS *NUMB* KO S1 subclone. (**A**,**E**) Migration assay. (**B**,**F**) Invasion assay. (**C**,**G**) Anchorage-independent colony formation. (**D**,**H**). Lactate production. The decreased oncogenicity and lactate production due to *miR-31* deletion was partially reversed by exogenous expression of *MCT1* or *MCT4*. The increased oncogenicity and lactate production due to *NUMB* deletion was partially reversed by the knockdown of *MCT1* or *MCT4* expression. Scr, scramble oligonucleotide; VA, vector alone. *ns*, not significant; *, *p* < 0.05; **, *p* < 0.01; ***, *p* < 0.001.

To address the effects of the *miR-31*-*NUMB*-*MCT1*/*4* axis in regulating tumor phenotypes and lactate production, the *miR-31*-deleted KO#51 subclone, *NUMB*-deleted S1 subclone, and parental SAS cells were studied. In KO#51 cells, the reduced migration, invasion, colony formation, and lactate production were rescued by *MCT1* or *MCT4* expression to varying extents (Figure 7A–D). The effects of *MCT4* expression on oncogenicity were particularly prominent. Likewise, the phenotypes and lactate production capability of the S1 subclone were reversed by the knockdown of *MCT1* or *MCT4* (Figure 7E–H). The effects of *MCT4* on invasion and colony formation were particularly prominent. These functional clues substantiate the existence of the *miR-31*-*NUMB*-*MCT1*/*4* regulatory axis in OSCC.

## 3. Discussion

A novel finding in the present work is that NUMB affected lactate production and glycolytic respiration in OSCC cells. In previous studies, *miR-31* was shown to target *SIRT3* to inhibit mitochondrial activity [7] and target *ACOX1* to disrupt the lipidome profile in OSCC [8]. Other studies reported that *miR-31* modulated the metabolic profile by targeting different types of cells [10,11,37]. As *NUMB* is the direct target of *miR-31* and several other oncogenic miRNAs [23,25,26], findings in this work substantiated that *miR-31* is an oncogenic miRNA that also modulates complex metabolic regulation in OSCC [7,8]. Inhibition of *NUMB* by *miR-31* also exerts both oncogenic and metabolic effects. We validated the efficacy of *miR-31* deletion using a double sgRNA-guided CRISPR/Cas9 cleavage approach [38]. This strategy can be further improved to achieve efficient *miR-31* blockade for in vivo tumor therapy.

Although *NUMB* has been known to affect complex controls on cellular functions, including oncogenesis and mitochondria [22,39], the current study employed *NUMB* deletion to gain insight into the function of *NUMB* in OSCC cells. The increased lactate production and the glycolytic metabolic switch appeared to coincide with the increase in MCT1/MCT4 protein levels in *NUMB*-deleted subclones. In line with these findings, the knockdown of *NUMB* and induction of endogenous *NUMB* expression also yielded results suggesting that *NUMB* regulated MCT1/MCT4 expression and lactate production. Since the knockdown of *MCT1*/*MCT4* also rescued the phenotypic and metabolic changes in *NUMB*-deleted cells, MCT1/MCT4 may modulate metabolic reprogramming and oncogenesis in addition to lactate flux [32,33,35]. The dual inhibition of MCT1/MCT4 ameliorated tumor growth [33]. In this study, we showed that NUMB concomitantly inhibited MCT1 and MCT4, which would inhibit neoplastic growth.

NUMB acts as an adaptor to promote polyubiquitination and induce degradation of various essential factors [14,16,19,20,21,22]. We identified the binding between Numb4 and MCT1/MCT4 in 293T cells and the increased polyubiquitination of MCT1/MCT4. Furthermore, MCT1/MCT4 protein abundance increased when *NUMB* was silenced in cells. Thus, the increase in lactate production following *NUMB* deletion is likely due to the increase in MCT1/MCT4 [29,30]. However, the role of *NUMB* on the mitochondrial machinery underlying the respiratory switch awaits further investigation. CD44, CD147, or β-integrin interact with MCTs to act as chaperones or functional patterners [40,41,42]. Although the entire NUMB protein was required for the binding of NUMB with mGluR5 [28], during transdifferentiation from mesodermal cells to endothelial progenitor cells, the splicing factor mediated the induction of *NUMB*_*S* isoforms to regulate Notch signaling [27]. The present study demonstrated, for the first time, the presence of Numb4 and MCT1/MCT4 complexes, which may trigger proteasome degradation, although the PTB and PRR domains were absent in Numb4 [12]. As NUMB does not possess ubiquitin ligase activity [18], further studies are required to elucidate the interaction sites in the NUMB protein and the mechanisms of ubiquitin ligase recruitment for degradation.

Although the different NUMB isoforms may be responsible for diverse cellular phenotypes [27], our previous studies demonstrated that isoforms Numb1 and Numb4 possessed comparable suppressor activity in OSCC cells expressing endogenous NUMB [23]. The present study further established that both Numb1 and Numb4 were equally suppressive in OSCC subclones whose *NUMB* genome had been deleted. The knockout model can be valuable to specifically evaluate the activity of each individual isoform in the disease process [27]. Our *NUMB* knockout approach adopted a solitary sgRNA-guided DNA cleavage and recombination. However, diverse and complex Indel patterns were induced around the cleavage site in the *NUMB* genome in subclones. This could be the reason why the faint NUMB signals were observed. Alternatively, a small fraction of functionally silent parental cells may be still present in the subclones. A faint signal may also be an artifact caused by the cross-reactivity of the antibody. As the suppressor role of *NUMB* is definitive, endogenous *NUMB* induction using the CRISPR/dCas-SAM system being tested in this study would be a potential option for cancer therapy.

Lactate is a crucial fuel source required for sustained energy support in tumor cells [32]. In addition to the modulation of lactate flux, the vital roles of MCT1/MCT4 in OSCC tumorigenesis were defined in this study. In agreement with a previous study [35], our database analysis also indicated the concurrent high expression of *MCT1* and *MCT4* in HNSCC, which defined poor patient prognosis. There were at least 70 genes whose aberrant expression highly correlated with *MCT1* expression in the TCGA HNSCC tumor cohort [33]. Although it is still not clear whether MCT-mediated oncogenic or metabolic shifts are due to the direct regulation of downstream effectors, they may interact with each other and also with partners, and disrupt metabolic homeostasis related to lactate flux [40,41,42]. Therefore, the inhibition of MCTs could be a promising therapeutic option [33,36].

## 4. Material and Methods

### 4.1. Cell Lines and Reagents

SAS and OECM1 OSCC cell lines along with 293T cells were cultured as previously described [1,2,25,43]. Small interfering RNA oligonucleotides (Table S1) and their scramble (Scr) controls were purchased from Thermo Fisher Scientific (Waltham, MA, USA). The doses of oligonucleotides have been validated as 60 or 120 nM. *TransFectin^TM^* Reagent (BioRad, Hercules, CA, USA) was used for transfection. Unless specified, all reagents were obtained from Sigma–Aldrich (St. Louis, MO, USA).

### 4.2. qRT–PCR Analysis

TRI reagent (Molecular Research Center, Cincinnati, OH, USA) was used to isolate RNA from cells. TaqMan miRNA assay kits (Apply Biosystems, Waltham, MA, USA) were used to quantify the expression of *miR-31, NUMB, MCT1*, and *MCT4* using *RNU6B* or *GAPDH* as internal controls (Table S2). The difference in gene expression between samples was calculated using the 2^−^^ΔΔCt^ method, where Ct is the threshold cycle [2].

### 4.3. Western Blot

Cell lysates were subjected to Western blot analysis using appropriate primary and secondary antibodies (Table S3). Signals of tested proteins were normalized to GAPDH to quantify expression levels [2].

### 4.4. Clustered Regularly Interspaced Short Palindromic Repeat (CRISPR)/Cas9 Gene Edition

The oligonucleotides were annealed to form double-stranded 5′ and 3′ sgRNAs to delete mature hsa-*miR-31-5*p (Table S4). Each sgRNA was cloned into the pU6-sgRNA.pPuro vector (National RNAi Consortium, Taipei, Taiwan) and co-transfected with the p5w-Cas9.pBsd vector (National RNAi Consortium) into cells (Figure S1A). After puromycin selection, single cells isolated by limiting dilution were expanded to obtain subclones [38]. qRT–PCR was used to detect *miR-31* expression. PCR products encompassing the deletion segment in cell subclones (Table S5) were cloned into the pHE vector (Addgene, Cambridge, MA, USA), and plasmid DNA from multiple bacterial colonies was sequenced to ascertain the presence of *miR-31* deletion in cell subclones [43].

The pAll-*NUMB*-Cas9-Ppuro vector, which co-expresses Cas9 and sgRNA targeting *NUMB* (Table S4), was obtained from the National RNAi Consortium (Figure S2A). A pSurrogate reporter vector (National RNAi Consortium) was used to confirm the effectiveness of the CRISPR/Cas9 system [43]. After selection with puromycin, cells expressing red fluorescence from mCherry were sorted and expanded to establish subclones. In addition, PCR products encompassing the deletion segment in cell subclones were cloned into a bacterial vector (Table S5), and plasmid DNA from multiple bacterial colonies was sequenced to ascertain the presence of *NUMB* deletion in cell subclones [43].

### 4.5. Induction of NUMB Activation Using Crispr-dCas9 SAM System

The potential sequence segments allowing dCas9 recognition within ~250 bp upstream of the *NUMB* transcription start site were predicted by the E-crisp (http://www.e-crisp.org/E-CRISP/assessed date: 20 September 2017) module [44]. Eight oligonucleotides containing sgRNAs that recognize these sequences were ligated into the sgRNA (MS2) cloning backbone (Addgene) to generate *SAM* constructs for promoter activation (Figure S3). The dCas9-SAM#6 construct was validated as the most potent construct for *NUMB* induction in pilot tests (Table S4).

### 4.6. Plasmid Construction and Overexpression

The coding sequences of *MCT1*, *MCT4*, and *ubiquitin* transcripts were amplified by PCR and cloned into pcDNA3.1(+) or pcDNA3.1(−) vectors for exogenous expression (Table S5). The vectors used for the overexpression of *Numb1* and *Numb4* variants were previously established by our group [23].

### 4.7. Phenotypic and Tumorigenic Assays

Cell proliferation, wound closure, migration, invasion, and anchorage-independent colony formation experiments were carried out according to previously published protocols [23]. For migration and invasion assays using a Transwell apparatus, cell growth was arrested by treatment with 1 μM hydroxyurea. Unless specified, migrated cells and invaded cells in 400X image fields and the colonies in 100X image fields were counted. For the induction of subcutaneous xenografts, 5 × 10^5^ cells were injected into the flanks of nude mice. Tumor volumes were calculated using the formula: volume = 0.5*ab*^2^; *a*, the longest diameter, *b*, the shortest diameter [2,43]. This animal study was approved by the Institutional Animal Care and Use Committee (IACUC) of National Yang-Ming University (IACUC approval no.: 1070503).

### 4.8. Co-Immunoprecipitation (Co-IP)

Anti-NUMB, anti-MCT1, or anti-MCT4 antibodies were conjugated with protein A magnetic beads according to the protocol provided by the supplier (Pierce™ Classic Magnetic IP/Co-IP Kit, Thermo Fisher Scientific). Mouse or rabbit IgG were used as negative controls (Table S6). After transfection with overexpression plasmids, 500 μg of cell lysate isolated from SAS or 293T cells were used as input for immunoprecipitation. After incubating input with pre-coupled beads, proteins on the beads were extracted with a sample buffer. All samples were boiled for 5 min before Western blotting analysis.

### 4.9. Measurement of Lactate Production and Mitochondrial Respiration

L (+)-lactate in culture medium obtained from equal numbers of cells was measured by a lactate colorimetric assay kit (BioVision, Milpitas, CA, USA). The mitochondrial respiration of cells was measured by a Seahorse XF24 Extracellular Flux system following the protocols provided by the supplier (Seahorse Bioscience, North Billerica, MA, USA). The oxygen consumption rate (OCR) and extracellular acidification rate (ECAR) were determined by a calculation software provided with the appliance [7].

### 4.10. Statistical Analysis

Data are shown as the mean ± SE. The Mann–Whitney test, *t-*test, and two-way ANOVA were performed. The GEO database was assessed through the website (http://www.ncbi.nlm.nih.gov/geo/assessed date: 28 February 2019). The genes in the HNSCC subset of the TCGA database were analyzed using UCSC Xena Functional Genomics Explorer (https://xenabrowser.net/assessed date: 24 July 2018), linear correlation analysis and Kaplan–Meier survival curves. *p* < 0.05 was considered statistically significant.

## 5. Conclusions

In conclusion, this study identified the presence of the *miR-31*-*NUMB*-*MCT1/MCT4* axis in mediating oncogenesis and metabolic switching, which implies that disruption of this cascade may intercept tumor pathogenesis and aerobic glycolysis.

## Supplementary Materials

The following are available online at https://www.mdpi.com/article/10.3390/ijms222111731/s1.

## Abbreviations

Crispr	Clustered: regularly interspaced, short palindromic repeats
GEO	Gene expression omnibus
HNSCC	Head and neck squamous cell carcinoma
OSCC	Oral squamous cell carcinoma
SAM	Synergistic activation mediator
TCGA	The Cancer Genome Atlas
