# Supplementary material for: miR-31-NUMB Cascade Modulates Monocarboxylate Transporters to Increase Oncogenicity and Lactate Production of Oral Carcinoma Cells
Source: Int J Mol Sci. 2021 Oct 29;22(21):11731. doi: 10.3390/ijms222111731 (PMC8584161; doi:10.3390/ijms222111731)
Supplement: Supplementary file 1 [file ijms-22-11731-s001.zip › ijms-1426589-supplementary.pdf]

Supplementary Tables

Table S1. siRNA and Scr control

Silencer™ siRNA Control No.1	Thermo Fisher Scientific	AM4611
Silencer™ *NUMB* siRNA	Thermo Fisher Scientific	13537
Silencer™ *MCT1* (*SLC16A1*) siRNA	Thermo Fisher Scientific	106943
Silencer™ *MCT4* (*SLC16A3*) siRNA	Thermo Fisher Scientific	106945

Table S2. Probes used for qRT-PCR

GAPDH	Thermo Fisher Scientific	Hs00266705_g1
miR-31	Thermo Fisher Scientific	002495
NUMB	Thermo Fisher Scientific	Hs01105433_m1
RNU6B	Thermo Fisher Scientific	001093
SLC16A1 (MCT1)	Thermo Fisher Scientific	4351372
SLC16A3 (MCT4)	Thermo Fisher Scientific	4331182

Table S3. Antibodies used in this study

GAPDH	Santa Cruz Biotech	sc-22233	Mouse	1:10,000
GLUT1	Abcam	ab32551	Rabbit	1:500
GLUT4	Abcam	Ab15311	Rabbit	1:500
HIF1α	BD Biosciences	610958	Mouse	1:1,000
MCT1	Santa Cruz Biotech	sc-365501	Mouse	1:500
MCT4	Santa Cruz Biotech	sc-376140	Mouse	1:500
NUMB	Abcam	ab14140	Rabbit	1:1,000
Ubiquitin	Invitrogen	13-1600	Mouse	1:1,000
anti-mouse-(HRP)	Millipore	AP124P	Goat	1:1,000
anti-rabbit-(HRP)	Millipore	AP132P	Goat	1:1,000

Table S4. Design of sgDNAs for CRISPR/Cas9 approach

	Sense oligonucleotide	Antisense oligonucleotide
miR-31 5' sgRNA	CACCGTAACTTGGAACTGGAGAGG	AAACCCTCTCCAGTTCCAAGTTAC
miR-31 3' sgRNA	CACCGTGCTGGCATAGCTGTTGAAC	AAACGTTCAACAGCTATGCCAGCAC
NUMB sgRNA	CACCGTGCCACTGATGTGGACGAC	AAACGTCGTCCACATCAGTGGCAC
NUMB dCas9-SAM#6	CACCGTTAGAAGTATGAAAGAGGGT	AAACACCCTCTTTCATACTTCTAAC

Table S5. Primers for PCR

Objective	Forward / Reverse primer (5' to 3')	PCR product (bp)
miR-31 deletion detection	AGCGGACACTCTAAGGAAGAC	WT: 281<br>Del: ~253
	GCACATACACAGCAATACACG	
NUMB indel detection	CTGAGGCTTGGTGAAGTTACG	~526
	CATGAAGCAGTGACAGATCCAG	
pcDNA3.1 (-) *MCT1*oe construct	GGCCTCGAGGCCACCATGCCACCAGCAGTT	1530
	GCGGGATCCTCATCAGACTGGACTTTCCTCCTCC	
pcDNA3.1 (-) *MCT4*oe construct	GGCCTCGAGGCCACCATGGGAGGGGCCGTG	1425
	GCGGGATCCTCAGACACTTGTTTCCGGGGTG	
pcDNA3.1 (+) *Ubiquitin* oe construct	CGCGGATCCATGCAGATCTTCGTGAAAACCC	249
	CCGGAATTCTAACCACCTCTCAGACGCAG	

Table S6. Antibodies used for immunoprecipitation

MCT1	Santa Cruz Biotech	sc-365501	Mouse
MCT4	Santa Cruz Biotech	sc-376140	Mouse
NUMB	Thermo Fisher Scientific	MA5-14897	Rabbit
Mouse IgG control	Millipore	AP106P	
Rabbit IgG control	Millipore	AP132P	

Supplementary Figures

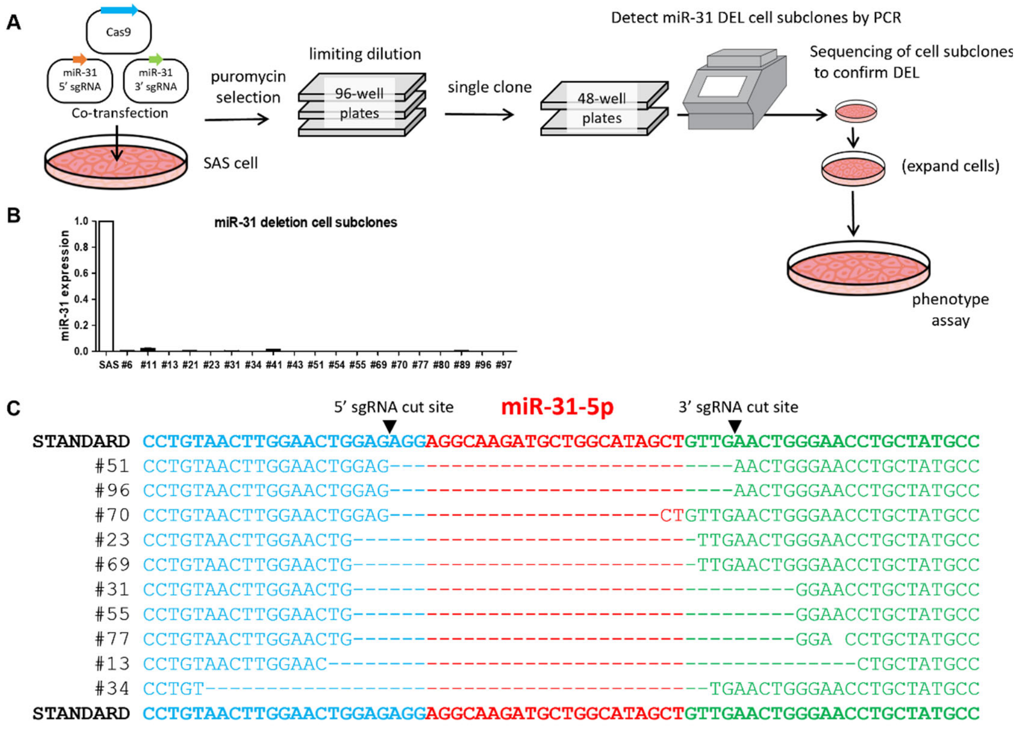

Figure S1. *miR-31* deletion. (A) 5'sgDNA and 3'sgDNA are designed to delete hsa-*miR-31*-5p. The procedures for the deletion of *miR-31*-5p are illustrated. (B) qRT-PCR analysis to detect *miR-31* expression. This reveals the almost complete absence of *miR-31* expression in 19 subclones acquired. (C) Sequencing reveals the truncations of various segments in 10 selected clones

A

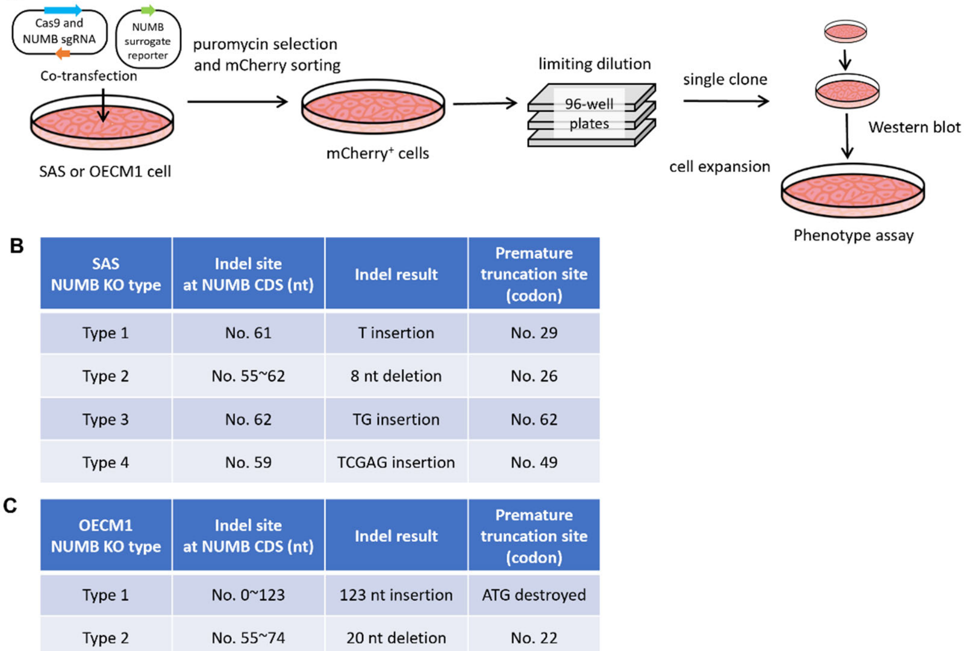

B

SAS NUMB KO type	Indel site at NUMB CDS (nt)	Indel result	Premature truncation site (codon)
Type 1	No. 61	T insertion	No. 29
Type 2	No. 55~62	8 nt deletion	No. 26
Type 3	No. 62	TG insertion	No. 62
Type 4	No. 59	TCGAG insertion	No. 49

C

OECM1 NUMB KO type	Indel site at NUMB CDS (nt)	Indel result	Premature truncation site (codon)
Type 1	No. 0~123	123 nt insertion	ATG destroyed
Type 2	No. 55~74	20 nt deletion	No. 22

Figure S2. *NUMB* deletion. (A) sgRNA is designed to delete *NUMB*. The selection, sorting, and population expansion procedures of cell subclones are illustrated. (B, C) Sequencing reveals the varying types of *NUMB* deletion or insertion in SAS and OECM1 cell subclones, respectively.

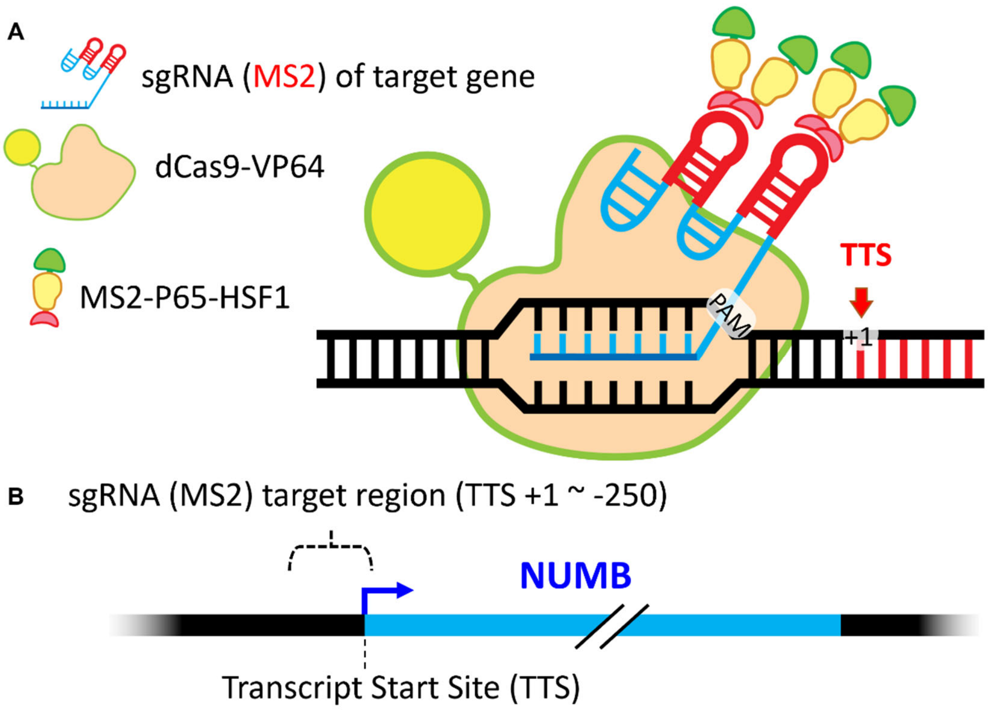

Figure S3. Synergistic activation mediator system for *NUMB* promoter activation. (A) Schema of components included in synergistic activation mediator (SAM) system. (B) Illustration of sgRNA targeting region in *NUMB* promoter.

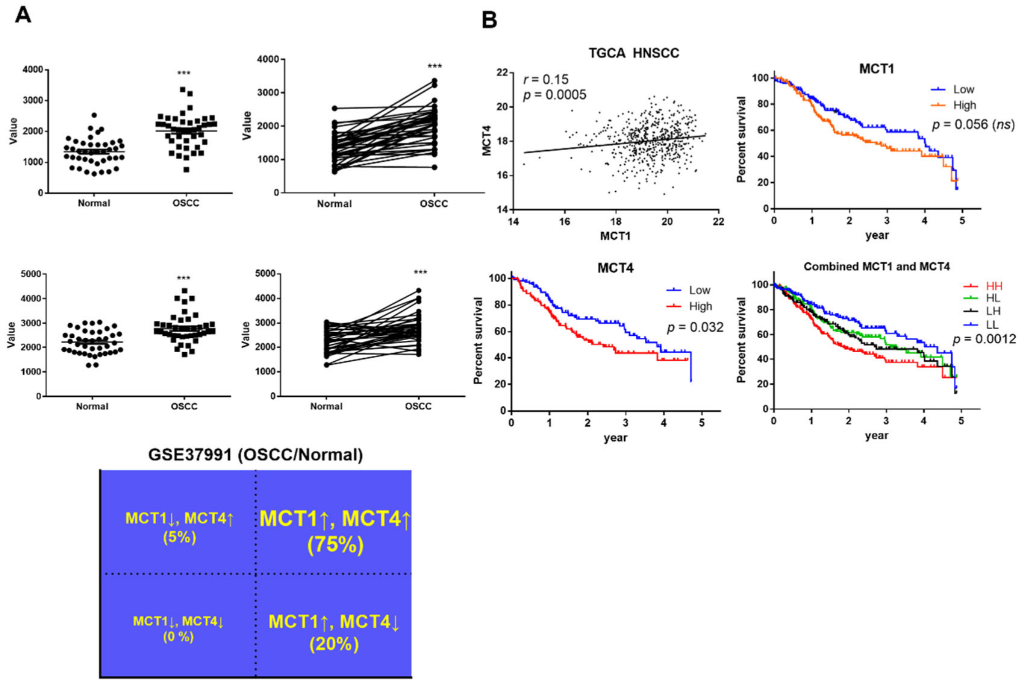

Figure S4. The *MCT1* and *MCT4* expression states in database. (A) GEO database GSE37991 *MCT1* (ILMN_1757052; upper) and *MCT4* (ILMN_2364022; middle). The un-paired *t*-test (left) and paired *t*-test (right) both show a significant upregulation of *MCT1* and *MCT4* in OSCC relative to matched normal tissue. A three-fourth of tumors exhibit the co-upregulation of *MCT1* and *MCT4* expression (lower). (B) TCGA HNSCC database. High correlation in the expression of *MCT1* and *MCT4* is noted in HNSCC (upper left). The high *MCT1* or *MCT4* expression defines the worse HNSCC prognosis (upper right and lower left, respectively). Tumors having both high *MCT1* expression and high *MCT4* expression define the worst prognosis (lower right). *ns*, not significant; ***, p <0.001.
